# Supplementary material for: Easily Attach/Detach Reattachable EEG Headset with Candle-like Microneedle Electrodes
Source: Micromachines (Basel). 2023 Feb 6;14(2):400. doi: 10.3390/mi14020400 (PMC9963435; doi:10.3390/mi14020400)
Supplement: Supplementary file 1 [file micromachines-14-00400-s001.zip › micromachines-2191483-supplementary.pdf]

# Supplemental Materials

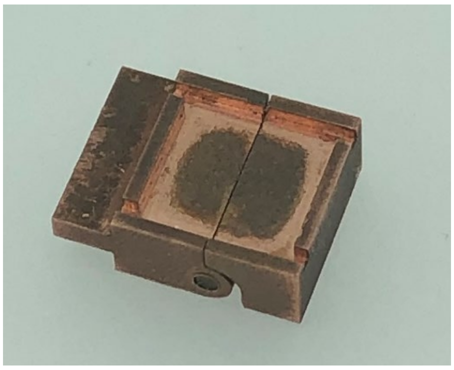

(**a**)

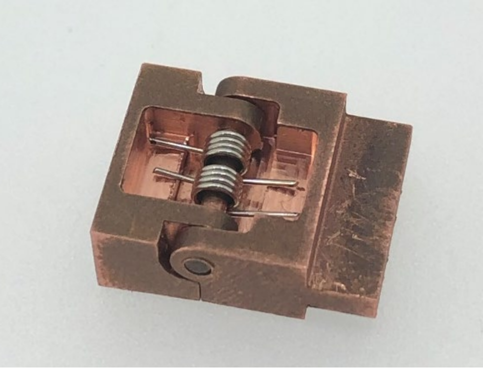

(**b**)

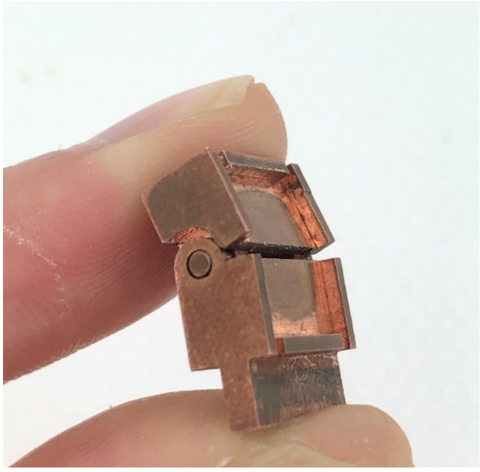

(**c**)

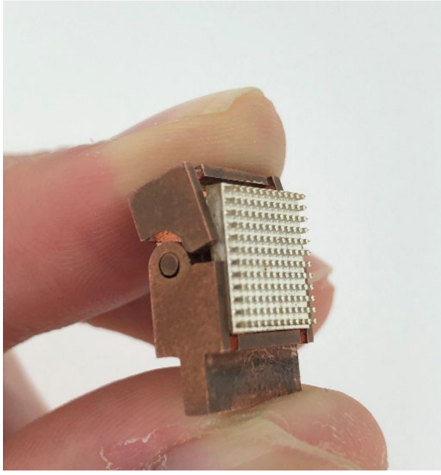

(d)

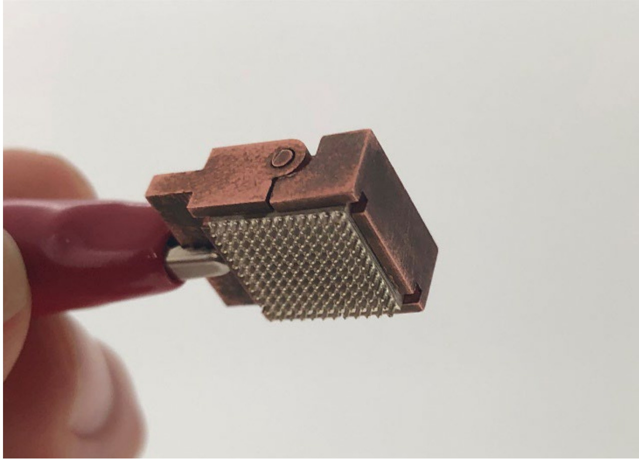

(e)

**Figure S1** Electrode holder made of copper. **(a)** Front and **(b)** back of the holder. The holder is composed of two parts, which are connected with a spring. **(c, d)** The holder can open and hold the CME. **(e)** The CME is supported by the holder tightly. The EEG acquired with the CME can be accessed via the holder.
